# Paclitaxel: Application in Modern Oncology and Nanomedicine-Based Cancer Therapy

**DOI:** 10.1155/2021/3687700

**Published:** 2021-10-18

**Authors:** Javad Sharifi-Rad, Cristina Quispe, Jayanta Kumar Patra, Yengkhom Disco Singh, Manasa Kumar Panda, Gitishree Das, Charles Oluwaseun Adetunji, Olugbenga Samuel Michael, Oksana Sytar, Letizia Polito, Jelena Živković, Natália Cruz-Martins, Marta Klimek-Szczykutowicz, Halina Ekiert, Muhammad Iqbal Choudhary, Seyed Abdulmajid Ayatollahi, Bekzat Tynybekov, Farzad Kobarfard, Ana Covilca Muntean, Ioana Grozea, Sevgi Durna Daştan, Monica Butnariu, Agnieszka Szopa, Daniela Calina

**Affiliations:** ^1^Phytochemistry Research Center, Shahid Beheshti University of Medical Sciences, Tehran, Iran; ^2^Facultad de Ciencias de la Salud, Universidad Arturo Prat, Avda. Arturo Prat 2120, Iquique 1110939, Chile; ^3^Research Institute of Biotechnology & Medical Converged Science, Dongguk University, Goyangsi, Republic of Korea; ^4^Department of Post-Harvest Technology, College of Horticulture and Forestry, Central Agricultural University, Pasighat, 791102 Arunachal Pradesh, India; ^5^Environment and Sustainability Department, CSIR-Institute of Minerals and Materials Technology, Bhubaneswar, 751013 Odisha, India; ^6^Applied Microbiology, Biotechnology and Nanotechnology Laboratory, Department of Microbiology, Edo University Iyamho, PMB 04, Auchi, Edo State, Nigeria; ^7^Cardiometabolic Research Unit, Department of Physiology, College of Health Sciences, Bowen University, Iwo, Osun State, Nigeria; ^8^Department of Plant Biology Department, Institute of Biology, Taras Shevchenko National University of Kyiv, Kyiv 01033, Ukraine; ^9^Department of Plant Physiology, Slovak University of Agriculture, Nitra 94976, Slovakia; ^10^Department of Experimental, Diagnostic and Specialty Medicine-DIMES, Alma Mater Studiorum, University of Bologna, Via San Giacomo 14, 40126 Bologna, Italy; ^11^Institute for Medicinal Plants Research “Dr. Josif Pančić”, Tadeuša Košćuška 1, 11000 Belgrade, Serbia; ^12^Faculty of Medicine, University of Porto, Porto, Portugal; ^13^Institute for Research and Innovation in Health (i3S), University of Porto, Porto, Portugal; ^14^Institute of Research and Advanced Training in Health Sciences and Technologies (CESPU), Rua Central de Gandra, 1317, 4585-116 Gandra, PRD, Portugal; ^15^Chair and Department of Pharmaceutical Botany, Jagiellonian University, Medical College, Medyczna 9, 30-688 Kraków, Poland; ^16^H.E.J. Research Institute of Chemistry, International Center for Chemical and Biological Sciences, University of Karachi, Karachi, Pakistan; ^17^Department of Pharmacognosy and Biotechnology, School of Pharmacy, Shahid Beheshti University of Medical Sciences, Tehran, Iran; ^18^Department of Biodiversity of Bioresources, Al-Farabi Kazakh National University, Almaty, Kazakhstan; ^19^Department of Medicinal Chemistry, School of Pharmacy, Shahid Beheshti University of Medical Sciences, Tehran, Iran; ^20^Banat's University of Agricultural Sciences and Veterinary Medicine “King Michael I of Romania” from Timisoara, Timisoara, Romania; ^21^Department of Biology, Faculty of Science, Sivas Cumhuriyet University, 58140 Sivas, Turkey; ^22^Beekeeping Development Application and Research Center, Sivas Cumhuriyet University, 58140 Sivas, Turkey; ^23^Department of Clinical Pharmacy, University of Medicine and Pharmacy of Craiova, 200349 Craiova, Romania

## Abstract

Paclitaxel is a broad-spectrum anticancer compound, which was derived mainly from a medicinal plant, in particular, from the bark of the yew tree *Taxus brevifolia* Nutt. It is a representative of a class of diterpene taxanes, which are nowadays used as the most common chemotherapeutic agent against many forms of cancer. It possesses scientifically proven anticancer activity against, e.g., ovarian, lung, and breast cancers. The application of this compound is difficult because of limited solubility, recrystalization upon dilution, and cosolvent-induced toxicity. In these cases, nanotechnology and nanoparticles provide certain advantages such as increased drug half-life, lowered toxicity, and specific and selective delivery over free drugs. Nanodrugs possess the capability to buildup in the tissue which might be linked to enhanced permeability and retention as well as enhanced antitumour influence possessing minimal toxicity in normal tissues. This article presents information about paclitaxel, its chemical structure, formulations, mechanism of action, and toxicity. Attention is drawn on nanotechnology, the usefulness of nanoparticles containing paclitaxel, its opportunities, and also future perspective. This review article is aimed at summarizing the current state of continuous pharmaceutical development and employment of nanotechnology in the enhancement of the pharmacokinetic and pharmacodynamic features of paclitaxel as a chemotherapeutic agent.

## 1. Introduction

As a result of the nonavailability of adequate treatment, cancer is nowadays becoming a major and life-threatening disease worldwide. Recent studies indicated that the number of cancer deaths globally exceeded the mortality rate of cardiac diseases [1]. It represents one of the greatest challenges of medicine today with the presumed increase in newly diagnosed cases in the near future and powerful impact at any stage of sociosanitary care [2]. General actions in prevention, early diagnosis, screening, and treatment of cancer have been questioned by its complex nature and mutability [3]. The occurrence of different tumour types may be attributable to several lifestyles and environmental factors such as radiation, infections, low physical activity, use of immunosuppressants, smoking, overweight, quality of diet, and alcohol consumption [1].

Over the past years, different cancer therapies have been developed. Fundamental treatments in cancer are surgery and radiation therapy, followed by various forms of specific treatments such as chemotherapy, immunotherapy, hormonal therapy, radiotherapy, and targeted therapy [1]. Even though therapies such as radiation and the application of standard chemotherapeutics possess significant tumouricidal potential, they usually kill normal cells besides cancer cells inducing that way serious hematologic toxicity and tissue damage in affected individuals [4]. Furthermore, chemotherapy shows narrow therapeutic benefits as a result of the occurrence of the intrinsic or acquired multiple drug resistance [5].

Compared to conventional therapies, targeted therapies with a multi-advantageous approach are much desirable [6]. They specifically stop the growth and expansion of cancer cells [7]. Their selective targeting profile is reflected in the fact that they achieve controlled release of the specific agent and its site-specific delivery and accumulation in tumour sites. As more efficient, targeted therapies reduce the demand for high doses of chemotherapeutics, that way, they generate less systemic adverse reactions [1].

The application of nanomedicine, which represents the integration of nanotechnology and medicine, in diagnosing, monitoring, and treating diseases has considerably enhanced the result of treatment of complicated and lethal diseases by retaining the therapeutic dose at the target site [8]. It includes the manipulation of the drug substance at the nanoscale which ultimately affects its physicochemical properties. Formulation approaches employing nanomedicines implicate the usage of sophisticated nanocarriers of natural and synthetic origin which shows immense potential to liberate therapeutics inside the body [1]. Numerous factors such as solubility, stability, biocompatibility, and biodegradability contribute significantly to the selection of nanomaterials for drug delivery demands [9]. Nanotechnology and nanoparticles provide certain advantages such as increased drug half-life, lowered toxicity, and specific and selective delivery over free drugs [10].

Nanomedicines that are presently used in clinical practice have been moderately effective in tackling the problems associated with drug development such as low solubility and inadequate circulation time [11]. Paclitaxel is a broad-spectrum anticancer drug from the group of the most commonly applied chemotherapeutic agents primarily for various solid tumours [12]. However, its application is significantly restricted as a result of limited solubility, recrystalization upon dilution, and cosolvent-induced toxicity [13]. The application of proper pharmaceutical carriers could overcome these limitations [14].

So far, paclitaxel nanoformulations have been efficiently utilized in clinical practice for the treatment of ovarian cancer, lung cancer, and breast cancer [15]. This narrative review article summarized the current state of the continuous pharmaceutical development and employment of nanotechnology in the enhancement of the pharmacokinetic and pharmacodynamic features of paclitaxel as a chemotherapeutic agent. As a novelty, this review highlights the therapeutic importance of paclitaxel nanoparticles in the targeted therapy of cancer cells in different types of tumours as well as data on their toxicity and safety.

## 2. Key Aspects of Targeted Therapy for Effective Cancer Management

Chemotherapy, lacking in specificity, is fundamentally based on the assumption that cancer cells have mitosis more frequently than normal cells, and for this reason, they can be more affected than healthy tissues [16]. However, this is not true for all cell types, thus, deriving the nonspecific toxicity of chemotherapeutic drugs for healthy tissues, characterized by cells with rapid proliferation rates, such as hair follicles, bone marrow, and gastrointestinal tract cells, generating the characteristic side effects of chemotherapy [17]. We can state that all chemotherapeutic drugs have two faces: pharmaceutical drugs at the right (for a specific case) concentration and toxic substances at higher concentrations. Nonspecific toxicity toward normal cells, narrow therapeutic windows, and the onset of multidrug resistance mechanisms [18, 19] have highlighted the limits of conventional cancer treatment with chemotherapeutics and the need to explore new ways to deal with the disease. Therefore, many researchers have been stimulated to develop new therapeutic approaches allowing a more targeted drug delivery to the cancer cell.

The idea to target cancer cells in a specific manner started from the hypothesis and studies of the Nobel Prize laureate Paul Ehrlich (in 1900), who postulated the “magic bullet concept”: an ideal drug that can go straight to its intended target on the cell surface [20]. Inspired by this concept, one of the most popular research approaches is based on the linking of a pharmacologically active molecule to carriers for selective delivery to target cells. These drugs could specifically target cancer cells to a larger extent with only a minimum impact on healthy cells.

Many different strategies have been exploited to carry toxic moieties. Different nanoparticles can facilitate drug delivery by both passive and active targeting mechanisms and highlight the potential advantages of utilizing nanotechnology within the field of cancer therapy [21]. Passive targeting is defined as the preferential accumulation of the drug in the tumour. Nanoparticles can be made from various materials including lipids [22]; inorganic materials such as gold, carbon, and iron oxide [23]; proteins [24]; and polymeric systems [25]. The accumulation and delivery of the drug are determined by the capability of the drug delivery system to overcome biological barriers and the inherent characteristics of the drug itself (e.g., size, material, and charge). In this strategy, drugs are enveloped in nanoparticles that can protect the therapeutic payload from blood degradation, with an improvement of their therapeutic index. However, due to the absence of specificity of these nanoparticles, it is possible to obtain only a limited reduction of some side effects. The size and the material of nanoparticles can affect their distribution and their intended goals.

Active chemotherapeutic drug targeting is aimed at blocking one or more specific hallmarks of cancer [26]: proliferative signalling, evading growth suppressors, resisting cell death, enabling replicative immortality, inducing angiogenesis, and activating invasion and metastasis [27]. This strategy is aimed at blocking specific pathways or cancer proteins, i.e., receptors, growth factors, and proteins related to apoptosis and angiogenesis, that are expressed in normal tissues but resulted in being overexpressed or mutated in cancer [28]. An essential requirement for this approach is that the target molecule is possibly confined to the cell population to be destroyed, or at least that it is not present on stem cells or other cell types essential for the organism's survival.

A type of active nanoparticles can be considered antibodies. They are the most utilized nanocarriers, due to their stability in blood and their avidity and affinity for the target antigen. To obtain these hybrid antibody-drug conjugates, the two molecules must be linked together. The linker should be stable in blood but cleavable within the target cells by specific enzymatic or chemical degradation, thus, releasing the drug in an active form that can kill the cancer cell [29]. In conclusion, although more than a century has passed from Erlich's initial idea, the concept of a “magic bullet” for cancer therapy has not yet faded.

## 3. Paclitaxel: Pharmacotherapeutic Synopsis

Paclitaxel was discovered as a consequence of a joint input from the US Department of Agriculture (USDA) and the National Cancer Institute (NCI) through the application of a plant-screening program for effective discovery of a novel drug that could be applied as an effective drug for the management of cancer diseases [30]. The program was conducted by the National Cancer Institute Plant directed by Jonathan Hartwell who is a chemist who deals with natural products in collaboration with a botanist from the US Department of Agriculture named Robert Perdue. They evaluated almost 15,000 medicinal plants globally as well as evaluated their crude extracts numbering approximately 115,000 to validate their effectiveness as an anticancer drug against cancer diseases for a duration that varies from 1960 to 1981. Moreover, the program involved in the selection of the active compound that was performed by NCI in 1981 showed that paclitaxel was the only active biological ingredient that fits into this category and met the standard that could be utilized for effective management of cancer diseases most especially from the clinical trials [31, 32].

Paclitaxel was derived from a medicinal plant, specifically from the bark of the yew tree *Taxus brevifolia* Nutt. which belong to the *Taxaceae* family. The medicinal plant was later transported from Gifford Pinchot National Forest to their headquarters at the US Department of Agriculture in Maryland in August 1962. This was performed by the US Department of Agriculture botanist Arthur S. Barclay [30, 31, 33, 34]. Also, the extract derived from the bark of this plant was established to possess a cytotoxic action that could affect the *in vitro* development of 9KB cell cultures responsible for the development of human oral epidermoid carcinoma [35, 36]. In the year 1990, a motion was raised to include the severely depleted *T. brevifolia* as among the number of endangered species in the Pacific Yew Act being passed that was signed into law that year to prevent the intellectual properties of the tree in the year 1992 [37]. The most active component was eventually identified through the help of Monroe Wall and a second person, Mansukh Wani, who worked in collaboration and with the help of the National Cancer Institute at the Research Triangle Institute to perform this action. The active constituent was named paclitaxel in the year 1967 [30].

Paclitaxel was shown to possess a great range of antitumour action after several *in vivo* screens carried out on tumours implanted in laboratory mice [31, 38]. The modes of action of paclitaxel were further established by Dr. Susan Horwitz. This experiment was performed at the Albert Einstein College of Medicine. Moreover, another milestone on paclitaxel that was documented establishes the capability of the plant to inhibit the development of tumour development most especially in the mammary tumour xenograft [30]. Also, Hartwell discovered that only half a gram of purified paclitaxel extract from the crude extract which contains 13 kilograms of dried bark contains the active component while other components are invaluable. This constitutes a factor that affected the investigation of paclitaxel on numerous cancers which mushroomed when carried out at clinical trials.

Furthermore, a great challenge that was encountered on the discovery of paclitaxel as an effective anticancer drug was that it has a high level of insolubility in an aqueous solution, but this problem was resolved through an enhanced formulation that entails Cremophor EL and ethanol. Cremophor EL has been identified as polyethoxylated castor oil that could be applied as a carrier for solubilization of hydrophobic drugs such as paclitaxel [39]. Moreover, one of the side effects of paclitaxel was its hypersensitivity reactions which limit their application using Cremophor EL as the drug vehicle, and this consequence affected any additional clinical trials due to the high number of deaths recorded. An extended and dawdling infusion was established to require 24 hours to minimize this severe adverse influence [40]. The application of paclitaxel was documented as a treatment option agent for the management of one of the most dangerous and common diseases called ovarian cancer. The scientist also affirms the efficacy of paclitaxel for the management of advanced breast cancer.

### 3.1. Paclitaxel: Chemical Characteristics

Paclitaxel has been recognized as a group of taxane drugs that could be referred to as diterpenoid pseudoalkaloid possessing an empirical formula of C_47_H_51_NO_14_, while 853.9 g/mol represents its molecular weight. There is the presence of 2 molecules having a homochiral ester side chain at C13 as well as the presence of a taxane ring having a four-membered oxetane side ring around C4 and C5 together with a homochiral ester side chain at C13. Moreover, the side chain present at C13 has been affirmed to perform an important function which represents the most effective portion that could join to the microtubules which play an important action in the stabilization of the tubulin bundles and triggers the disorganization of the microtubules available in a guanosine triphosphate- (GTP-) independent manner. This led to the prevention of cell penetration by inhibiting the cell cycle most especially at the metaphase/anaphase borderline. It has been established that an ester side chain and an intact taxane ring are very crucial for cytotoxic action [41, 42].

### 3.2. Paclitaxel: Mechanism of Action

It has been stated that the application of paclitaxel as an anticancer drug possesses the ability to attack microtubules. Microtubules possess a cylindrical hollow body shape in nature with a diameter that varies from 25 to 30 nm, which is made up of numerous polymers of tubulin which are in dynamic equilibrium together with tubulin heterodimers which entails beta- and alpha-constituents of the protein subunits [43, 44].

The fabrication of tubulin and the collation of microtubules happened at the prophase of mitosis and the G2 phase. It has been stated that microtubules exist in the state of dynamic steadiness most especially with the subunit tubulins such as *β* and *α* placed in a head-to-tail arrangement. The plus ends tend to be faster when compared to the minus ends which is slower at the other end. Moreover, it has been documented that the length of the microtubule remains unaltered under steady-state conditions.

Furthermore, it has been stated that the minus part that constitutes part of the microtubules are usually joined to the centrosome [45], but the plus ends use the cytoplasm and relate with the cellular structures [46, 47]. Dr. Horwitz established that comparison of paclitaxel to vinca alkaloids showed that they can hinder cell separation by enhancing the joining together of stable microtubules particularly from the 𝛽-tubulin heterodimers as well as the prevention of the process of depolymerisation which plays a crucial function toward the inhibition of the G2/M phase of the cell cycle, death of the cell, and prevention of cell replication [48, 49].

Paclitaxel also possesses the capability to join precisely in a rescindable manner to the N-terminal 31 amino acids available at the beta-tubulin subunit present in the microtubules relatively to tubulin dimers [49, 50]. Moreover, paclitaxel can enhance microtubule formation *in vitro* when subjected to cold temperatures (4°C) without the presence of GTP [48]. Furthermore, investigation on the proper knowledge of the molecular modes of action of the microtubule development has established that paclitaxel possesses the capability that could enable the cell to avoid drug toxicity, which might lead to the development of a more resistant drug which might affect its application as a chemotherapy drug [47], and the investigation has discovered that the microtubule dynamics does not have any relationship to the cell division.

It was discovered that the application of treatment to the following mutant cell lines which entails Tax 18 and Tax 11-6 when tested at concentrations of 50 to 100 nmol/L decreases the microtubule dynamics instead of restoring behaviour to normal. Under normal conditions, there should be an enhancement of microtubule assembly to additional normal levels which enables the cell to grow into a normal cell [47]. It has also been discovered that the presence of a lower concentration of paclitaxel reduced dynamics, which did not show any effect on the degree of microtubule separation. The presence of a greater therapeutic concentration stimulates cell division which greatly prevented the disinterestedness in the mutant cell lines which enable them to be geared back to their normal level. This could be linked to the potential of paclitaxel to prevent microtubule separation instead of its capability to inhibit or prevent microtubule changing aspects, which could be established through the help of live cell imaging which shows that microtubule detachment that occurs at the centrosomes could be a crucial factor that constitutes the development of microtubule fragments, a process that could be overturned by paclitaxel [47].

Also, it has been established that the presence of paclitaxel, whenever it is present in a lower concentration, most especially when available in nanomolar concentrations, could prevent the process involved in the depolymerisation of microtubules, but their presence at a very high concentration could enhance the mass of microtubules and their number. Therefore, improving the steadiness of microtubules could prevent their nonfunctional action through the inhibition of the process involved in the removal of the microtubule minus ends, most especially from the centrosomes, when compared to the plus end [47, 51].

Also, based on the apoptotic influence of paclitaxel performed on the modes of action carried out during a weekly route experiment, it was shown that paclitaxel possesses a capability to stimulate the apoptotic modulator genes which shows that paclitaxel is independent of microtubule maintenance. This might be linked to the regulation of the process of the transcription of numerous genes which includes inflammation, DNA-damage response proteins, apoptosis, and proteins or cytokines that play a crucial role in the regulation of cellular proliferation. The rate of cell apoptotic influence of paclitaxel has been established to depend on the dose and the time of exposure. For example, the application of a concentration containing 10 nM which has been exposed for 12 hours may trigger cell death through the induction of the S phase without mitotic arrest. It has been established that the application of a dose containing ≥9 nM paclitaxel could stimulate Raf-1 activation which regulates cell death control. Also, the dose containing ≥9 nM showed the nonappearance of Raf-1 kinase participation, but the cell death induction is regulated through the action of p21 and p53 [52, 53]. Moreover, it was affirmed that the presence of the same dose when applied at 24 hours could lead to cell death and generate an irretrievable mitotic arrest [54]. The presence of p53 as a significant tumour suppressor plays a crucial role in the prevention of cell death and cell proliferation while mutation could happen in more than 50% of human cancers; however, in a normal cell, it has been stated that the damage of DNA in p53 could stimulate the G1 cell cycle arrested by p21 to enhance the process involved in the repair mechanisms or cell death which naturally prevent the production of genetically transmuted cell clones.

It has been established that a well-designed p53 signalling route is paramount to the stimulation of cancer cells and all the chemical therapeutic techniques involved in its prevention. It has been shown that paclitaxel action does not depend on p53 status which may be mutated or silent as well as constitute a major factor that decreases its chemoresistant action [55–58].

Also, it has been demonstrated that paclitaxel possesses the capability to stimulate the action of many signal-transduction routes that are related to pre-cell death signalling. The route related to paclitaxel entails the following which was P38 mitogen-activated protein, stimulator of transcription factor route, TLR-4-dependent pathway, Janus kinase signal transducer, c-Jun N-terminal kinase, and nuclear factor kappa B [55–58].

Furthermore, the stimulation of cell death has been documented through a mitogen-activated protein kinase route leading to phosphorylation of the pre-cell death protein Bax and Bad, Bcl2 decreased cell death, phosphorylation of Bcl2, and Bad and Bax enhanced cell death, all of which constitute a regulatory protein that plays a crucial role in programmed cell death. It has been shown that the stimulation of proinflammatory proteins and cytokines could lead to the production of the immunomodulatory influence of paclitaxel when applied to a lower dose and the stimulation of apoptosis when applied to a higher concentration. The alteration that exists in these pathways is accountable for the high level of resistance to paclitaxel [59–62].

Also, it has been established that paclitaxel possesses the capability to exhibit a greater angiogenic inhibitory action when performed weekly [63, 64], while the application of paclitaxel in a murine evaluation shows that it portends the tendency to decrease the new vessel formation when applied at a lower, noncytotoxic concentration containing 0.3 and 6 mg kg^−1^ day^−1^ in mice by decreasing the manifestation of vascular endothelial growth factor [65, 66]. Also, other modes of action through which paclitaxel exhibits an effect involves the postponement of the time of administration and may play a crucial role in patients suffering from cancers becoming more resistant to drugs when administered on a 3-weekly conventional schedule [67, 68].

The application of paclitaxel as an anticancer has been affirmed that this drug could stimulate the presence of hydroperoxide through the triggering of the action of the nicotinamide adenine dinucleotide phosphate oxidase [69, 70]. Also, the synergetic effect of paclitaxel together with inhibitors of glucose such as 2DG and 2-deoxy-D-glucose as well as substances that contain hydroperoxide metabolisms such as L-buthionine-S,R-sulfoximine have been established to prevent the cancer cell inhibition of the breast through the action of the H_2_O_2_-stimulated metabolic-induced oxidant in the management of breast cancers [70].

## 4. Paclitaxel: Nanomedicine-Based Cancer Therapy—Recent Advances and Challenges

Nanomedicine could be stated as the utilization of nanotechnology most especially in the medical field for the management and diagnosis of life-threatening diseases such as cancer [71]. It has been observed that the application of nanomedicine gives a more precise and accurate detection and encourages the effectiveness of the drugs applied by minimizing the adverse influence related to standard therapeutics [72]. The application of nanomedicine has been observed in the treatment of cancer such as trajectory delivery of nanodrugs, nanopharmaceuticals, and nanoanalytical disparity chemicals in a research laboratory and animal prototypical research [73].

Also, the discovery of numerous new nanomedical products during the clinical trials and their eventual distribution into the commercial market has grown tremendously over the years, most especially their application for the treatment of cancer and their application in the diagnosis of cancer diseases [74].

This enhances the therapeutic effects of some active compounds through several novel techniques that have been evaluated that could lead to enhanced retention of drugs most especially when carried out *in vitro* studies. Typical examples of such involve the incorporation of high molecular substance agents such as hydrogels and nanoparticles [75–80].

The utilization of nanotechnology has been recognized as an effective technique that could deliver nanodrugs [81, 82]. It has been established that nanodrugs possess the capability to buildup in the tissue which might be linked to enhanced permeability and retention as well as enhanced antitumour influence possessing minimal toxicity in normal tissues [83]. The enhanced permeability and retention influence could be linked to certain features of solid tumours such as immature lymphatic drainage, hyperpermeability of tumour vessel walls, and partial vascular architecture [84]. Leveraging on all these concepts, numerous types of nanodrugs have been fabricated for the management of several types of cancer diseases [85].

It has been observed that repetitive intraperitoneal introduction of paclitaxel together with simultaneous systemic chemotherapy has been established to show a high level of effectiveness for the treatment of peritoneal metastases obtained from gastric cancer. However, the process involved in the biochemical alterations has been established to influence the bioavailability of intraperitoneally administered paclitaxel.

In 2017, Kitayama et al. [86] utilized a xenograft repetitive intraperitoneal model utilizing human gastric cancer cells. Fluorescein-conjugated paclitaxel was repetitively administered intraperitoneally, and the intratumour circulation of paclitaxel was assessed utilizing fluorescein microscopy. The result obtained indicates that paclitaxel was observed to unswervingly penetrate up to numerous hundreds of micrometers most especially superficially through the repetitive intraperitoneal administration immediately after intraperitoneal inoculation. The coadministration of 5% nonanimal stabilized hyaluronic acid enhances paclitaxel infiltration and inhibited the growth of peritoneal metastases when compared to paclitaxel alone. Moreover, it was discovered that the solubilization of paclitaxel along with an amphiphilic polymer possessing 2-methacryloyloxyethyl phosphorylcholine and n-butyl methacrylate led to the development of a micellar formation which has a diameter that ranges from 50 to 100 nm in diameter. Additionally, it was discovered that the administration of nanomicellar paclitaxel (paclitaxel-30W) improved tumour penetration and prevented the development of peritoneal metastases when compared with paclitaxel solubilized with cremophor-ethanol (paclitaxel-Cre). Furthermore, repetitive intraperitoneal injection of NK105, which represent another nanomicellar paclitaxel, prevents the development of subcutaneous tumours, such as together with peritoneal metastases, when compared to the unadventurous paclitaxel-Cre in the same murine model. Their study showed that the repetitive intraperitoneal paclitaxel injection could enhance the penetrability potential of peritoneal metastases which could serve as techniques for the management of peritoneal metastases. The alteration of drug-using nanotechnology could improve the level of infiltration of peritoneal metastases leading to enhanced clinical effectiveness.

It has been shown recently that the prevention of liver metastases derived mainly from lung and breast cancers have exhibited little effectiveness. This might be linked to the fact that liver metastases are not properly vascularized, which prevented the capability of transport therapeutics mainly through the systemic circulation to lesions.

In 2016, Tanei et al. [87] utilized a system that could introduce nanoparticle albumin-bound paclitaxel (nAb-paclitaxel) into a nanoporous solid multistage nanovector material that improves the movement of the therapeutic agent into the tumour microenvironment. This might also trigger their relationship with the liver macrophages. Also, it was shown that nanoporous solid multistage nanovector enablement could enhance nAb-paclitaxel effectiveness and persistence in mouse models of lung liver and breast metastasis. Nanoporous solid multistage nanovector-nAb-PTX also amplified the buildup of paclitaxel and the level of nanoporous solid multistage nanovector available in the liver, most especially the macrophages, but the level of the paclitaxel available in the blood remained unaltered after injecting nAb-paclitaxel and nanovector-nAb-paclitaxel.

The *in vitro* study carried out showed that the macrophages administered with nanoporous solid multistage nanovector-nAb-paclitaxel maintained their viability and internalized, maintained, and discharged meaningfully advanced amounts of paclitaxel when compared to the treatment with nAb-paclitaxel. The cytotoxic effects of the liberated paclitaxel were also affirmed in the tumour cells cultured together with the supernatants of macrophages applied with the nanoporous solid multistage nanovector-nAB-paclitaxel. Their study established that conveying nAb-paclitaxel to liver macrophages most especially when it occurs within the tumour microenvironment could produce an enhanced therapeutic reaction most especially in patients having metastatic liver cancer without any sign of enhancing systemic side influence.

The effectiveness of chemotherapy which is derived from radiation methods has been discovered to often undergo swift clearance and off-target toxicity. The application of a drug delivery system and the control of therapeutic drugs in a controlled release manner have been observed to enhance the effectiveness of the therapeutic efficiency of drugs most especially those that contain small molecules.

In 2018, Yang et al. [88] synthesized 2 new nanodrugs using oxidative and reductive techniques for the fabrication of paclitaxel prodrugs which can quickly conjugate with albumin in an *in vivo* study. It was discovered that albumin could serve as a natural means of transporting the nanodrugs to the tumours which might be linked to the increase in the retention and permeation influence. It was observed that paclitaxel could induce the liberation of reactive oxygen species/glutathione cancer cell microenvironment. Their experiment indicated that the bioresponsive prodrug approach shows the effectiveness of chemotherapeutic effectiveness when tested in an *in vivo* assay which may affect the management of cancer diseases.

It has been stated that multifunctional nanoparticles could be applied to enhance the management of index and minimize the side influence of antitumour drugs.

In 2019, Lei et al. [89] synthesized a highly biocompatible drug which is a multifunctional nanoparticle incorporated with gemcitabine, paclitaxel, and folic acid using self-assembly toward targeted and affected cancer cells. The result obtained from the transmission electron microscopy established that the fabricated nanoparticles which are multifunctional FA targeting nanoparticles (MF-FA NPs) are spherical with encouraging enhanced structural stability most especially when present in an aqueous solution. Furthermore, it was observed that the MF NPs and the MF-FA NPs showed proliferation inhibition when applied to the breast cancer cell 4T1 when compared to pure drugs. The result of the *in vivo* antitumour experiment carried out established that the nanoparticles could enhance the anticancer effects in comparison with the synthetic drug. Moreover, MF-FA NPs showed an enhanced greater tumour growth inhibition when compared to the MF NPs which might be linked to the presence of some specific influence directing FA to cancer cells. Therefore, the innovative MF-FA NPs might be utilized as an important nanodrug for the management of breast cancer.

Paclitaxel has been recognized as one of the major active and potent chemotherapeutic drugs that have been discovered to show higher inhibitory activities against different types of cancer such as ovarian, breast, and lung cancers. It has been discovered that paclitaxel has a lower solubility in water; therefore, paclitaxel is normally formulated in a mixture containing a combination of dehydrated ethanol and cremophor EL (50 : 50, *v*/*v*) which constitutes a combination referred to as taxol. Moreover, several side effects as a result of utilizing ethanol and cremophor EL have been reported. Therefore, there is a need to develop an alternative taxol formulation [90, 91].

Therefore, the application of encapsulation techniques that utilize paclitaxel which is a biodegradable and nontoxic nanodelivery system will go a long way toward the prevention of the drugs from dilapidation most especially during the process of circulation and thereby prevent the body from any available toxic or adverse effect. Moreover, it will also show a more enhanced patient compliance, minimize its toxicity, enhance its circulation half-life, and enhance pharmacokinetic profiles. Moreover, it has been affirmed that the nanoparticle-based delivery systems have several merits such as improved permeability and retention influence most especially for the targeting of passive tumours which shows that they are promising carriers that could increase the therapeutic index and reduce of the total side effect of paclitaxel [12]. In 2017, Wang et al. [92] wrote a comprehensive review on the expansion of paclitaxel drug delivery systems and evaluate the design values supporting each delivery stratagem.

The application of paclitaxel albumin-bound nanoparticles (Abraxane®) has been approved by the Food and Drug Administration for the management of breast and lung cancer. Moreover, numerous new paclitaxel nanoparticle formulations have been tested in clinical trials. Therefore, in 2013, Ma and Mumper [12] wrote a comprehensive review on the application of paclitaxel nanodelivery systems such as nanoparticles, polymeric nanoparticles, cyclodextrin, lipid-based formulations, nanocrystals, polymer conjugates, carbon nanotubes, and inorganic nanoparticles.

In 2016, Paciotti et al. [93] carried out the fabrication of different types of thiolated paclitaxel analogues which has been identified as a new nanomedicine program that was formulated toward effective development of nanomedicine involving paclitaxel and gold nanoparticles, most especially for tumour targeted drug delivery. The initial assessment of the novel nanomedicine entails 27 nm gold nanoparticles, thiolated polyethene glycol (PEG-thiol), numerous thiolated paclitaxel analogues, and tumour necrosis factor-alpha (TNF-*α*). There is a higher demand for an effective and more effective drug without any side effects. This has led to the discovery of innovative paclitaxel formulations, which have been subjected to clinical trials.

In Table 1, the type of nanoformulation, anticancer activity, and mode of delivery of paclitaxel are shown.

## 5. Cancer and Targeted Therapy

### 5.1. Cancer and Its Triggering Factors

Cancer is a specific disease where cells are formed that cannot divide without control, are immune to death, and can attack other tissues [113]. It is considered one of the leading causes of death in the world [114]. According to previous research, in 15-20% of cancer cases, autoimmunity in the same tissue or organ site, chronic inflammation, or infection was also diagnosed [115, 116]. In such cases, inflammation may appear earlier than tumour formation. The most prominent examples include inflammatory bowel disease (IBD), chronic hepatitis, helicobacter-induced gastritis, or schistosoma-induced bladder inflammation that increases the risk of colorectal cancer (CRC), liver cancer, stomach cancer, or bladder cancer, respectively [117].

Factors that can predispose to cancer include environmental factors that can cause some or all of the chronic inflammation, sometimes of low intensity [118]. In these cases, the inflammation that is present may develop with or before the tumour. Concerning the host, these factors might be systemic or site and organ specific. The development of lung cancer and mesothelioma may be caused by the formation of pneumonia and the respiratory tract by inhaling tobacco smoke and asbestos [119, 120]. Inflammation caused by obesity, hyperglycemia, and excessive lipid accumulation is usually systemic and may increase the risk of various types of cancer such as liver, pancreatic, colon, breast, and other malignancies [121, 122].

Type II diabetes is classified as one of the independent cancer risk factors. It is believed to lead to cancer formation through obesity-induced inflammation and obesity-related tissue injury. Obesity is an increasingly common problem in westernized countries, so it is important to determine the mechanism by which obesity and inflammation associated with it affect the growth of cancer. This is to alleviate the consequences of metabolic diseases common in society. In the late stages of the tumour, systemic inflammation can also take place, for instance, due to tobacco smoke, obesity, and bacterial-product-induced inflammation. Neutrophils are activated, and their extracellular trap formation functions to promote breast cancer metastasis into the lungs [123].

#### 5.1.1. Inflammation and Oncogenesis

**Figure 1:**
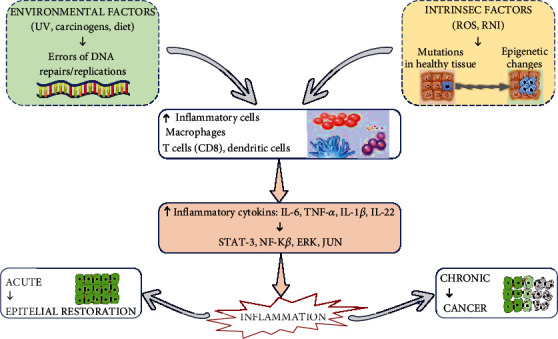
A diagram regarding the correlation between different factors (environmental, intrinsic), inflammation processes, and the effects on tissues. *Abbreviations*: ROS—reactive oxygen species; RNI—reactive nitrogen species; IL-6—interleukin 6; TNF-*α*—tumour necrosis alfa; IL-1*β*—interleukin 1*β*; IL-22—interleukin-22; STAT3—signal transducer and activator of transcription 3; NF-*κ*B—nuclear factor kappa-light-chain-enhancer of activated B cells; ERK—extracellular signal-related kinase.

Tumour initiation occurs as a result of two independent events:

1. One leads to the accumulation of mutations and/or epigenetic alterations of genes and signalling pathways involved in tumour suppression (inactivation) and oncogenic pathways (activation). They were usually associated mainly with environmental factors (e.g., UV, carcinogens, and/or variable radiation), as well as with errors in DNA repair and replication (Figure 1). It is worth noting that inflammatory reactions also involve mechanisms contributing to the accumulation of mutation and various epigenetic changes in adjacent epithelial cells.

Chronic intestinal inflammation leads to the accumulation of mutations in Tp53 and other cancer-related genes in intestinal epithelial cells [124–127] and can trigger tumour formation even without additional extrinsic mutagens being present [128].

Interestingly, evolutionary inflammation has the potential to induce mutation and DNA damage, an example being IL-22, which induces DNA damage response (DDR) gene expression to counter possible inflammation-induced genotoxic injury [129]. Besides, cytokine signalling (i.e., IL-6, TNF-*α*, and IL-1b) produced by inflammatory cells activates epigenetic machinery in epithelial cells, including DNA and histone modification components (Dnmt1, Dnmt3, and telomeric silencing disruptor 1 (DOTL1)), microRNA- (miRNA-) and long noncoding RNA- (lncRNA-) modulating oncogene expression levels, and tumour suppressors [115].

The net outcome of such epigenetic changes is proposed to be the same as inactivating mutations in tumour suppressors and activating mutations in oncogenes and can potentially be achieved in a “bi-allelic” manner at the same time. There are many cases where stem cells are considered to be “cells of origin” for cancer, and therefore, inflammatory processes can cause dedifferentiation of postmitotic epithelia into tumour-initiating stem-like cells [130].

Tissue damage that may result from chronic inflammation leads to the weakening of the barrier, and thus cells may be exposed to carcinogens, e.g., environmental carcinogens, or leads to the presence of stem cells close to active inflammatory cells producing genotoxic compounds. In another case, cancer occurring in, e.g., the colon, is rich in microbes and inflammation may be amplified thereby causing changes in qualitative characteristics of epithelial-adhesive microbiota, enriching the content of species harbouring genotoxic gene products, such as colibactin in some strains of *E.coli*, which may be capable of inflicting mutations in host cells [8, 131] which may be capable of inflicting mutations in host cells.

(2) The development of transformed and/or malignant clones, however, should be accompanied by their growth into a frank tumour, a cycle to which inflammatory pathways can make a major contribution. Inflammation is induced by reactive oxygen (ROS) and nitrogen (RNI) species which are produced by macrophages and neutrophils. This contributes to the development of mutagenesis and the accumulation of mutations in healthy tissue (Figure 1).

For example, cytokine receptor signalling in mutated cells can converge in the activation of prosurvival pathways, especially mediated by NF-*κ*B, STAT3, and other types of signalling [132, 133] thereby increasing the probability of survival of transformed clones or allowing proliferation. Specifically, early inflammatory signalling may be needed in tumour initiation. Some of the cancer cells may have not yet evolved a full-scale, auxiliary TME that needed growth factors to support tumours. There is also an inflammation-driven cell survival problem regarding cancer immune surveillance and tumour removal process of mutated and stressed cells [134, 135].

Signals activating STAT3 shield epithelial cells from CD8 T cytotoxic cell attack [136, 137] and IFN signalling, while normally anticytokine, enhances the expression of T cell fatigue, causing molecular programmed death-ligand 1 (PD-L1) in the transformed epithelium, which is recognized by T cells. Inflammatory signs may be used to the same goal, to improve fitness and decrease the expression of “stress ligands” on cancer cells needed for appropriate identification [138–140], and inflammation and injury cause cell turnover in tissues, providing space for the development of malignant clones [141].

As per the study, the tendency of inflammation to induce tumourigenicity leads to tumour progression. As per the translational standpoint, tumour growth can be driven by inflammation, and inhibition of inflammation can inhibit tumour growth and result in further progression [142]. The insight information widens the opportunity for early detection of cancers and shedding light on metastatic seeds spreading out even if distantly established. Multiple preclinical animal models have been instrumental to reveal the significant mechanisms associated with inflammation and cancer. Conceptually, there are many interdependent molecular and cellular mechanisms at play. First, inflammatory agents similar to tumour initiation may function as direct growth factors for rising tumours. Moreover, inflammatory factors in the tumour microenvironment (TME) is important in the formation of cell plasticity, as a result of which it influences tumour growth in three separate mechanisms [143].

Studies of NF-*κ*B inactivation in myeloid cells have demonstrated for the first time that inflammation may play a role in tumour promotion. Ablation of the nuclear factor-*κ*B (IKKb) kinase inhibitor resulted in a reduction in tumour enlargement in a colitis-associated cancer model [144]. In reality, NF-*κ*B regulates cytokine expressed particularly in immune cells which promotes the survival and expression of chemokines by acting on epithelial and tumour cells which is necessary for the recruitment of the tumour microenvironment (TME).

Genetic and pharmacological inactivation of NF-*κ*B-dependent cytokines reduces tumour growth due to activating other monogenic pathways in epithelial and cancer cells, including STAT3, extracellular signal-regulated kinase (ERK), JUN, and Tyr kinase receptor (TKR) [133, 145–148].

### 5.2. Cancer and the Need for Anticancer Drug Use

Cancer cells may be targeted at DNA, RNA, or protein at the molecular level; at the organelle or nucleus at the cell level; and at the endothelium and extracellular matrix at the tissue level according to the action mechanism of anticancer drugs [149]. Most traditional chemotherapeutic agents interact with cancer cell DNA, while monoclonal antibodies are directed against proteins or the endothelium and extracellular matrix [150]. Chemotherapy by traditional drug administration is the most common cancer treatment, but it involves several issues, including low drug solubility, low precision, high toxicity, and low therapy index [113, 151].

The platinum-based family of anticancer medication is composed of cisplatin (CDDP), carboplatin, oxaliplatin, and nedaplatin. These are antineoplastic drugs by blocking DNA replication and are used to treat a range of cancers including testicular cancer, cancer of the ovaries, cancer of the breast, and cancer of the bladder and lung [152]. Trabectedin (Yondelis®, ET-743), a semisynthetic tetrahydroisoquinoline alkaloid originally extracted from the marine tunicate *Ecteinascidia turbinata*, was the first marine anticancer agent to be approved for soft tissue sarcoma patients in the European Union [153, 154].

Among the clinically approved polyketides, ribulin mesylate (E7389) can be distinguished, its primary source of origin being the marine sponge *Halichondria okadai*. This compound is an analogue of halichondrin B that acts as a non-taxane microtubule dynamics inhibitor. Since 2010, compound E7389 has been approved by the FDA for the treatment of liposarcoma and breast cancer [155]. Bryostatin 1 is sourced from sponges and tunicates; it is a polyketide isolated from *Bryozoan bugula neritin*. This compound is active against many carcinomas [156]. Cytarabine is another compound (Ara-C) which is synthetically derived from spongothymidine and isolated from the marine sponge *Cryptotheca crypt*. This compound is a nucleoside that has already been licensed and marketed and has been used primarily in leukaemia [157]. Gemcitabine is a nucleoside analogue (specifically, a fluorinated cytarabine derivative) that could be used in cancer drug development [158, 159] and is used in numerous carcinomas, such as non-small-cell lung cancer, pancreatic cancer, prostate cancer, and breast cancer [157]. Doxorubicin is a metabolite of *Streptomyces petitcetius*. DOX is used as an antineoplastic agent in the treatment of fluid and solid cancers including breast cancer leukaemia. Unfortunately, DOX usage is correlated with toxicity that may result in extravasation, diarrhoea, vomiting, haematopoietic repression, alopecia, and cardiotoxicity [160].

Paclitaxel (PTX) in the taxane family of chemotherapeutics and herbal diterpenoid pseudoalkaloid is a white crystalline powder with a lipophilic nature. It has an empiric form C_47_H_51_NO_14_ and a molecular weight of 853.9 g/mol [161]. It demonstrates low solubility in water and melts at a temperature of 217 to 2°C [162].

PTX consists of two molecules, that is, a taxane ring with a four-piece oxetane side ring at positions C-4 and C-5, and a homochiral ester aspect chain at position C-13. The aspect chain at C-13 plays a crucial role as an active element that attaches to the microtubules, which stabilizes the tubulin bundles and activates the disassembly of microtubules in the guanosine triphosphate residue in an independent environment. As a result, cellular proliferation is disrupted by halting the phone loop at the periphery of metaphase/anaphase and by developing nonaccomplished chromosomes, which eventually contributes to the stability of the microtubule dynamics. A comprehensive lookup has shown that a broken taxane ring and an ester side chain are necessary for the development of cytotoxic action [41, 42].

PTX is an antitumour drug that activates microtubules. Microtubules contain cylindrical hole bodies that are 25–30 nm in diameter and include dynamic structured tubulin polymers along with heterodimers of tubulin molecules (comprising 5-007 as well as *β* protein subunits) [163]. The cytotoxic activity of PTX has been correlated with the stabilization of microtubules [164]. Microtubules play an important role in the formation of mitotic spindles at some stage in the process of mobile division. It is important to preserve the cell structure, motility, and cytoplasmic motion within the cells. The development of tubulin as well as the assembly of microtubules takes place at some point in the G2 segment and the mitosis prophase. Microtubules are in complex equilibrium with their tubulin subunits 5-007 and *β*, arranged in a head-to-tail manner with preferential faster growth (plus ends) at one end and slower boom (minus ends) at the other hand. Tubulin is used as a target factor for the cytotoxic activity of PTX, of which tumour cells in the mitosis segment have been arrested to cause tubulin affiliation to microtubules [48, 165].

### 5.3. Modifiable and Nonmodifiable Factors in Cancer Therapy

Cancer risk factors are those factors that increase a person's risk of developing cancer due to certain reasons. In most cases, the root cause of cancer development cannot be given, which causes one person to get sick and the other not. However, certain risk factors may increase a person's chances of developing cancer. The risk factor may be categorised as follows: modifiable cancer risk factors including the lifestyle and health behaviour of a person (e.g., consumption of tobacco products, alcohol, and food items that increases obesity); nonmodifiable cancer risk factors including the genetics of the family, their age, gender, race, and ethnicity; and environmental risk factors including exposure to chemical substances, radiation, infectious diseases, or any other agents which may cause cancer (Figure 1) [118, 166, 167].

It is also understood that for a particular type of cancer, different risk factors may apply. That means a complex interaction of multiple factors is playing together for a person's risk of developing cancer.

#### 5.3.1. Nonmodifiable Factors of Host

*(1) Age*. Age is a great risk factor for the development of cancer. It is being said that cancer cells are present in the human system. When and how it will express is a matter of concern and seems to be related to age. Cancer risk factors are often in age-old people as compared to the younger generation. For example, in the US, pancreatic cancer usually occurs between the ages of 40 and 80 years [168]. In India, the condition is slightly different. This incident of pancreatic cancer starts rising in the 5th decade and is prominent in the 6th decade [169].

*(2) Gender*. The incidence and mortality rate of various cancers are often related to sex disparities. Sex differences are one of the most significant findings in cancer epidemiology [170]. In most cases of cancer development, men are more susceptible than women, e.g., especially in haematological malignancies. Similar gender differences can be observed in nonmalignant diseases including autoimmunity, attributed due to hormonal (like estrogen, progesterone) or behavioural differences. The mortality of cancer is reported more in men than in women, especially for lung, colorectal, and stomach cancers [171, 172]. Female-specific cancers such as breast, ovarian, and uterine corpus and male-specific cancers such as prostate cancer are showing a high mortality rate. Men have a 34% higher risk of death than women in the case of melanoma cancer [173]. Antitumour effects of paclitaxel [174] that can interfere with cell division by depolymerizing cytoskeletal microtubules was reported in a female patient showing 20% lower elimination of paclitaxel than in a male patient [175].

*(3) Ethnicity*. Several risk factors can increase the chance of developing cancer in human beings. One of them is race as a risk factor for developing cancer. Race is a recognized risk factor for most cancer progression. Statistics analysis reveals that certain racial groups are more prone to some types of cancer than other racial groups around the globe. For instance, in the US, African-Americans are more susceptible to pancreatic cancer than Caucasians, and the lowest incidence was visible in Asian-American and Pacific Islanders [176]. The higher incidence in African-Americans is attributed and related to modifiable risk factors such as diet, vitamin D insufficiency, and alcohol consumption [177]. Asian patients appear to have less vulnerability and a better survival rate to cancer risk than Americans [178].

*(4) Blood Group*. The blood group of human beings can be discussed for cancer risk. ABO blood type is associated with the risk of all cancer types. However, the incidence of cancer risk varies according to the type of cancer. As compared with blood type A, blood type B is less susceptible to cancer risk factors [179]. Blood type B and AB have a lower risk of gastrointestinal cancer and colorectal cancer. Blood type B is likely to have a lower risk of stomach cancer and bladder cancer while blood type AB is more susceptible to liver cancer [179].

#### 5.3.2. Modifiable Risk Factors

*(1) Dietary Factors*. Lifestyle and dietary consumption play a significant role in risking cancer development. Nearly 30-40% of all cancers can be prevented by changing the diet and lifestyle [180]. The consumption of low-fibre food, concentrated sugars, refined flour products, and red meat and an imbalanced intake of omega 3 and omega 6 fatty acids puts one at more risk to develop cancer [181]. Intake of vegetables and fruits and other antitumour seeds will lower the risk of cancer development. Vegetables like cruciferous, allium, and broccoli contain sulforaphane, and these are beneficial in protecting and preventing against cancer. Some other diets which are useful to protect and prevent against cancer development may include selenium, folic acid, vitamin B-12, vitamin D, chlorophyll, and antioxidants. When a good guideline diet is properly taken, it is likely to reduce 60-70% of breast cancer, colorectal cancer, and prostate cancer, and even 40-50% in the case of lung cancer, and other similar reductions in cancer development [180].

*(2) Alcohol*. Alcohol is the common term for ethanol or ethyl alcohol. It is usually derived from rice, wheat, maize, grapes, etc., through the process of fermentation by yeast. It is being utilized as an important component in some medicines, mouthwashes, and other household products. There is a strong consensus from scientific reports that the consumption of alcohol causes different types of cancers, and it is one of the high risks for cancer development [182]. Consumption of alcohol is a known human carcinogen. From much evidence, it is indicated that consumption of alcohol regularly causes a higher risk of alcohol-associated cancer in a later phase of one's life [183]. A total of 3.5% of cancer deaths in the US are due to alcohol consumption [184]. Numerous studies have examined whether consumption of alcohol is a risk factor prominently causing different cancers which include head and neck cancer [185], esophageal cancer [186], liver cancer [187], and colorectal cancer [188].

*(3) Smoking*. Smoking is dangerous to health. Smoking will lead to tobacco-related cancer (TRC). According to WHO, it is estimated that one out of two young people who becomes a chain smoker will lead to TRC [189]. In India, the risk of developing cancer in between the age groups of 35 and 70 years due to the consumption of tobacco-related products was high in males (4.75%) as compared to females (2.16%). The total cancer risks due to tobacco consumption are around 45% in males and 20% in females [190]. The relation between smoking and lung cancer was first identified by a US surgeon in 1964. Subsequently, different other cancers like acute myeloid leukaemia (AML), cervix cancer, oesophagus cancer, kidney cancer, larynx cancer, trachea cancer, oral cancer, and pancreas cancer were also developed from smoking.

*(4) Infections*. After dietary factors and tobacco-related factors, infectious disease factors represent the third cause for cancer development worldwide. Several chronic viral papillomaviruses (several HPV types), herpesviruses (EBV and KSHV), polyomaviruses (SV40, MCV, BK, and JCV), and hepadnaviruses (HBV); RNA viruses, such as flaviviruses (HCV), defective viruses (HDV), and retroviruses (HTLV-I, HTLV-II, HIV-1, HIV-2, HERV-K, and XMRV); bacterial (*H. pylori*, *S. typhi*, *S. bovis*, *Bartonella*, and *C. pneumoniae*) and protozoan infections (*P. falciparum*); nematodes (*S. haematobium*, *S. japonicum*, *S. mansoni*, *O. viverrini*, *O. felineus*, and *C. sinensis*); and other microorganisms have been reportedly linked with human cancer development affecting different anatomical sites of the body. Infectious disease and parasitic disease claimed 10% of the population of cancer patients in the US [191], 10-20% in the UK, 3.6% in France, and 29.4% in China [192]. In the world, cancer that developed due to infectious diseases is estimated to be 15.6% in 1990 [193], 17.8% in 2002 [194], and 16.1% in 2008 [195]. Most of the viruses linked with the risk of cancer infestation can be passed from one person to another through the blood or other body fluids. One can lower the risk of cancer development by getting vaccinated.

### 5.4. Cancer-Targeted Therapy and Personalized Medicine

Traditional chemotherapy is based on the fact that the drug kills normal and cancerous cells. It is not only affecting the cancerous cells but also affects other surrounding cells. The targeted therapies are also termed precision medicine or personalized medicine which works differently from traditional therapies [196].

The idea of precision medicine is not new, but the recent advancement in technologies, materials, drugs, and medicine have helped to boost this area of research. Targeted therapies are applied only to that target cancer cells without hampering other normal cells in the body. The targeted therapies are emerging and most wanted technologies acquired by an oncologist for better diagnosis and treatment of cancer patients by looking to the current trends of increasing cases [197]. They are designed to interfere with the cellular activities of targeted cells by stopping the function of the gene. In some cases, the drug will attach to the molecules directly and stopping their metabolic activities. By doing so, the cancer cell will stop performing its normal metabolic activities thereby decreasing the growth and spread of the cancer cells. It is being called personalized medicine because of the patient' tumour cells. Every cancer patient has a unique molecular profile and different mutational mechanisms. The mutation of the cancer cell may vary according to the patient and the type of mutation. This means that certain types of drugs that works for one patient with lung cancer may not work for another. For instance, immune-oncology is a kind of immunotherapy that uses the patient's immune system to fight against cancer cells. Sometimes, this kind of therapy allows the doctor to selectively treat the patients based on their genetic history. This system of therapy is affected by three components which include biological, psychological, and social factors. Targeted therapies can be given in the form of pills or infusions in the bloodstream as per the requirement. The recent advancement in nanomedicine accumulation with curcumin is an effective therapy for targeted delivery in cancer cells [198].

#### 5.4.1. Antibodies as Targeted Therapies

Antibody exploitation in cancer therapy has become a major strategy for most of the oncologists to treat the patient due to the ability of antibodies binding to the specific primary and metastatic cancer cells. They have a high affinity toward targeted cells and produced antitumour effects through the following:

- Complement-mediated cytolysis and antibody dependent
- Cell-mediated cytotoxicity (naked antibodies)
- Focused delivery of radiation
- Cellular toxins (conjugated antibodies) [199–201]

In the US, the FDA has approved the use of 8 different anticancer therapeutic antibodies to increase the availability of drugs to the general public, and the details were provided by Ross et al. in 2004 [202].

Studies have shown that the use of the mouse monoclonal antibodies does not bring the expected results due to low overall potency of naked mouse antibodies as anticancer drugs, poor tumour cell penetration of antibodies, small radioisotope and toxin conjugate production, and the development of human antimouse antibodies (HAMAs) [203]. There are also partially humanized antibodies known to reduce HAMA responses and other related problems. Later on, human antibodies were developed based on murine sources and recombinant DNA technologies [204]. Modern antibody therapy is capable of reducing cytotoxicity of the drugs and other protein toxins that may create the problem.

## 6. Paclitaxel and Other Drugs: Emphasis on Synergistic and Potentiation Reactions

Paclitaxel is a representative of a class of diterpenes taxanes, which nowadays are widely used as a chemotherapeutic agent against various types of cancer. Another name of paclitaxel in use is taxol [205]. The source of paclitaxel is the Pacific yew tree (*Taxus brevifolia*). Initially, the class of taxanes was isolated from natural sources and later obtained by artificial synthesis [206]. The common hazel plant *Corylus avellana* shells and leaves have been reported to be a source of paclitaxel and other taxanes [207].

Taxanes are difficult to synthesize because of their numerous chiral centres—paclitaxel (or taxol) has 11 of these. Paclitaxel (Taxol) and docetaxel (Taxotere) are known to be used as chemotherapy agents [208]. Docetaxel is an efficient alternative in the treatment of patients with metastatic breast cancer for whom previous treatment has not worked [209]. The effect of docetaxel monotherapy, which consists of a 1-hour infusion every 3 weeks is comparable or more effective than that of doxorubicin, paclitaxel, and fluorouracil plus vinorelbine [210]. Cabazitaxel is a compound that has been classified by the FDA to treat hormone-refractory prostate cancer [211].

Due to the limited solubility in the water of taxanes, drug formulation is the difficult therapeutic potential of the taxanes paclitaxel (Taxol) and docetaxel (Taxotere), which can be limited by encumbrances faced by anticancer drugs such as toxicities, acquired multidrug resistance, and *de novo* refraction [205]. Due to their toxicity, both paclitaxel and docetaxel can predispose to and/or cause drug-induced lupus erythematosus, which most commonly manifests as subacute cutaneous lupus erythematosus [212–214]. To decrease the toxicity, some chemotherapeutic agents used the potential of synergistic reactions. Synergy is a mechanism by which the actions of certain substances together bring greater benefits and effects than the sum of their monoeffects.

The synergistic effects can be obtained between herbal products and conventional drugs or biochemical compounds [215]. In the in vitro model of non-small-cell lung cancer, it was claimed that there was synergism between paclitaxel and naturally occurring diet-based flavonoid fisetin. Studies have shown the ability to stimulate mitotic catastrophe and autophagic cell death [216]. The experimental studies of synergistic antitumour effect of *α*-pinene and *β*-pinene with paclitaxel against non-small-cell lung carcinoma showed morphological changes characteristic of apoptosis-like chromatin condensation and fragmentation of the nucleus [49].

Discodermolide is a polypropionate-based specific secondary metabolite isolated from the sea sponge. Discodermia dissolute in the past years was found to stabilize microtubules [217], and its synergistic potential for paclitaxel (Taxol) efficacy has been studied. The experimental studies for evaluation of drug combination of discodermolide and taxol indicated drug-induced aneuploidy enhancing effect rather than mitotic arrest in human ovarian cancer cells and in an in vivo model of ovarian carcinoma. Furthermore, in an animal model of ovarian carcinoma, the studied discodermolide and paclitaxel combination may suppress angiogenesis and induce regression of tumours [218]. Another secondary metabolite which belongs to the terpenes class was found to potentiate the lethality of paclitaxel via ROS-mediated mechanisms, and it was described that gelomulide K, which is a caspase-independent cell death-inducing agent, synergizes with paclitaxel on breast cancer cells and also has low toxicity to healthy cell treatment with gelomulide K-activated PARP-1 hyperactivation, AIF nuclear translocation, and cytoprotective autophagy, which relate to the intensification of ROS production [219].

The synergistic interaction of the conventional drug gemcitabine (brand name Gemzar) and paclitaxel by modulating acetylation and polymerization of tubulin in non-small-cell lung cancer cell lines have been shown and that the combination allows gemcitabine with paclitaxel to synergistically suppress tumour growth [220].

The other known conventional antifungal drug rapamycin (sirolimus) is able to potentiate the effects of paclitaxel in endometrial cancer cells through induction of apoptosis, inhibition of cell proliferation, acetylation of tubulin, and potentially increasing polymerization [221].

The antiparasite conventional drug praziquantel (brand name Biltricide) could greatly enhance the anticancer efficacy of paclitaxel resulting in a more pronounced inhibition of tumour growth compared with either drug alone in a mouse xenograft model [222].

## 7. A Brief Overview of Clinical Trials

Paclitaxel was produced as a natural material derived from the plant into a commercially viable semisynthetic drug that was developed to meet the increase in the demand for this drug [36]. Rapid advances in biomedical research have stimulated the development of nanomedicine and over the last 15 years, the number of studies employed to address its application in cancer therapy significantly increased [9, 223]. Due to the complexity and heterogeneity of the tumour environment, the distribution of nanomedicine and its interaction within the tumour area has shown considerable challenges [224].

The positive outcome of nanoparticles in clinical trials is conditioned with various factors:

- Stability and time spent by the particles in the circulation
- The capacity of the particles to pass physiological barriers and reach affected anatomic sites
- Bioavailability of particles at the affected area
- The safety profile of particles

Upgrading these features should help to achieve greater efficacy of nanomedicines, subsequently facilitating their promotion into standard cancer treatment [225].

Nowadays, there are numerous immunoconjugates under clinical trials. The great interest in the field of institutional investigators and pharmaceutical companies is also demonstrated by the increasing number of patented immunoconjugates, and many researchers agree that immunoconjugates will likely become important actors in cancer therapy in the foreseeable future. This research is today particularly interesting due to the availability of a new generation of mAbs, such as chimeric or humanized molecules, that are already at the clinical stage [226]. The development of combination therapies may result in new effective options for the treatment of cancer, and the knowledge of the mechanism of action of the substances is mandatory to design proper effective protocols.

Paclitaxel anticancer activity was established in a clinical trial after 22 years after it was discovered in the year 1984. Also, the first clinical trial was reported in 1987 by Wiernik et al. [40]. Paclitaxel was documented to have cytotoxic action most especially when carried out at the clinical trial during a trial that was performed on ovarian cancer. It was finalized that only 30% of ill people suffering from platinum-resistant ovarian cancer diseases showed a positive response to the treatment of paclitaxel whether partially or completely [227].

Some other clinical trials performed in the year 1994 showed that the drug exhibited a more inhibitory action against cancer diseases. The Food and Drug Administration finally approved this effective paclitaxel for utilization for the cure of breast cancer diseases. Paclitaxel is now applied when combined with other drugs such as carboplatin and cisplatin and as a single drug for the management of several cancer diseases [228]. The trial of the paclitaxel in patients with neck and head cancer, advanced ovarian cancer, and metastatic melanoma led to stability in these diseases [229–231].

The efficacy and clinical safety of albumin-bound paclitaxel (Abraxane®) as nanoparticles has been demonstrated in numerous clinical trials in various types of cancers. Thus, data available from 106 patients included in two open-label, single-arm studies and from 454 patients treated in a randomized phase III comparative study supported the efficacy of Abraxane in metastatic breast cancer [96, 232]. Another multicenter, multinational, randomized, open-label study was conducted, in which 861 patients compare the combination of Abraxane/gemcitabine with monotherapy with gemcitabine as first-line treatment in patients with metastatic pancreatic adenocarcinoma. There was a statistically significant improvement in progression-free survival in patients treated with Abraxane/gemcitabine compared to gemcitabine monotherapy, with a mean increase in survival of 1.8 months. A multicenter, randomized, open-label study was performed in 1052 patients with non-small-cell lung cancer in Stage IIIb/IV, in which no chemotherapy was administered. The study compared Abraxane in combination with carboplatin with the solvent-based paclitaxel in combination with carboplatin for first-line treatment in patients with non-advanced small-cell lung cancer. Patients in the Abraxane/carboplatin group had a significantly higher overall response rate larger compared to patients in the control group [233, 234].

## 8. Toxicity and Safety Aspects

Chemotherapeutic drugs for cancer management are accompanied by undesired adverse effects especially causing damage to other tissues of the body. Therefore, the application of nanotechnology for targeted drug delivery and biocompatibility and biodegradability offers an amazing opportunity to enhance the efficacy of cancer drugs while diminishing the toxic effects at the same time.

Paclitaxel (PTX) is a broadly used anticancer drug for a variety of cancers including breast, ovarian, gastric, and lung cancer [161]. The use of PTX clinically is reduced because of the concerns of toxicity such as low solubility, poor cancer cell selectivity, and speedy removal from the body. Likewise, PTX is associated with unwanted adverse effects such as hair loss, neurotoxicity, genotoxicity, hepatotoxicity, hypersensitivity, cardiac toxicity, allergic reaction, and repression of bone marrow [235].

The cancer patient's essence and worth of life is negatively affected because of the PTX-associated toxicity. Hence, this reduces the actual dose necessary for the beneficial effects of the chemodrug. The improvement of the mode of administration or delivery of PTX alongside exceptional anticancer potential with a good safety profile is necessary [236].

The application of nanotechnology in the field of biomedicine especially for the targeted drug delivery to a specific tissue of interest has gained research attention and clinical use, particularly for cancer treatment. Nanotechnology has been used to reduce toxicity and improve the safety and potency of PTX through the application of nanomaterials for efficient and effective delivery of the nanoformulated PTX drugs to the carcinoma cells and tissues [237]. Many biocompatible and biodegradable polymer-based nanoparticles have been used for drug delivery purposes [238].

### 8.1. Nanoparticle Albumin-Bound Paclitaxel (Nab-PTX)

**Figure 2:**
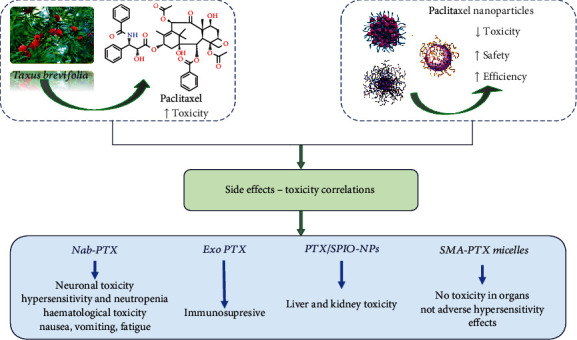
Summarized scheme regarding side effects of PTX nanoformulations.

The nab-PTX chemo-drug has been documented to possess limited safety issues compared to paclitaxel. However, nab-PTX is associated with neuronal toxicity (Figure 2). Furthermore, nab-paclitaxel is beneficial more than Crepaclitaxel, since it has a tremendous diminished possibility of hypersensitivity and neutropenia, and quick improvement of peripheral neuronal toxicity upon cessation of treatment [239]. Zong et al. have also reported the toxicity associated with nab-PTX chemotherapy to include haematological toxicity such as low neutrophil and leukocyte counts as well as elevated alanine aminotransferase and aspartate aminotransferase activities which were corresponding but higher in number than what was observed in the paclitaxel-treated group. Furthermore, nausea, vomiting, fatigue, and diarrhoea were also seen in the nab-PTX group [96]. Interestingly nab-PTX was reported to be cheap for the management of breast cancer when compared to other traditional taxanes because it reduces the toxic side effects and cost of management of any ensuing hospitalization [240, 241].

### 8.2. Bovine Milk-Derived Exosomal Formulation of Paclitaxel (ExoPTX)

Exosomes derived from milk is a feasible option because of their simplicity thereby enabling good mass production, quite comparable distribution in the biological system when administered through the oral route, and well-suited to the intracellular environment. Milk-derived exosomes possess innate defensive capability because of their antacid characteristics. Hence, they can serve as a great oral drug delivery system for cancer drugs such as PTX [98]. One of the most important functions of milk-derived exosomes is that they enhance the ease of drug usage. Milk-derived exosomes were shown to be immune-competent without any significant alterations in the spleen size, haematological parameters, and bone marrow. Therefore, ExoPTX did not have adverse effects on the immunological system of the body, unlike PTX which causes alteration in the responses of the immune system through the reduction in white blood cells. The toxic immunosuppressive effect associated with intravenous PTX administration has been completely taken cared of by the ExoPTX oral administration (Figure 2). The improvement offered by ExoPTX will enhance patient's experience and minimize the cost of cancer care [98].

### 8.3. Paclitaxel and Superparamagnetic Iron Oxide-Loaded PEGylated Poly(lactic-*co*-glycolic Acid)- (PLGA-) Based Nanoparticles (PTX/SPIO-NPs)

The understanding of the polymeric nanoparticles used for diagnosis and therapies is very crucial especially when it comes to the elucidation of their toxicity [242]. Since PTX/SPIO-loaded PLGA nanoparticles store up in the hepatic tissue, then it is quite important to ensure that they do not have any adverse effects. PTX/SPIO-NPs have been demonstrated to be safe for the liver because there was a histomorphological alteration in the hepatocytes of the PTX/SPIO-NP-treated mice. Furthermore, PTX/SPIO-NPs did not result in any elevation in levels of blood urea nitrogen, aspartate aminotransaminase, or alanine aminotransferase when compared with the control (saline-treated group), signifying that the nanoparticle does not have toxic effects on the liver and kidneys. Furthermore, there was no significant alteration in the body weight and creatine myocardial band (CK-MB) of the PTX/SPIO-NP-treated mice and the control showing the safety of the NPs in the heart. Therefore, PTX/SPIO-NPs will offer a good anticancer treatment [102].

### 8.4. Toxicity Analysis of SMA-PTX Micelles in the Orthotopic Colon Cancer Model

**Figure 3:**
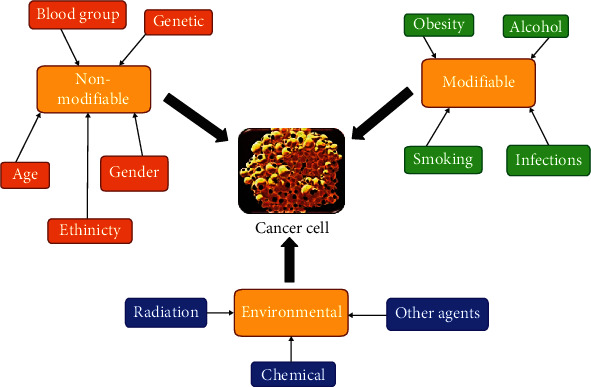
Risk factors for cancer development.

Treatment with SMA-PTX micelles did not alter the bodyweight signifying enhanced broad health of the mice. Furthermore, the SMA-PTX micelle-treated mice also had similar organ weights when compared with the control showing that there is no toxicity in the organs. Likewise, the SMA-PTX micelle group did not have any alterations in the alkaline phosphatase activity and creatinine levels indicating that there was no renal and hepatic tissue toxicity as a result of the treatment (AHC, 2015). Furthermore, SMA-PTX micelle nanoformulation is soluble in water and possesses no adverse hypersensitivity effects which are associated with PTX treatment. Interestingly, SMA-PTX micelle nanoformulation did not lead to any alteration in the anticancer effects of PTX. Therefore, the SMA-PTX micelle nanoformulation will offer an efficacious and nontoxic oral drug delivery. SMA-PTX micelle nanoformulation led to improved anticancer activity against colorectal cancer when compared with PTX [108] (Figure 3).

## 9. Overall Conclusions and Future Perspectives

Our work proves the usefulness of nanomedicine in cancer therapy. A special example is paclitaxel, which is a broad-spectrum anticancer drug. Scientific studies have confirmed its action against ovarian, lung, and breast cancer.

The review focused on the chemical structure of paclitaxel, its difficulties in application, and various nanoparticles containing paclitaxel—its usefulness and prospective toxic effect. It has been confirmed that the importance of nanomedicine is increasing in cancer therapy due to the reduced toxicity and improved safety and potency of such compounds as paclitaxel through the effective delivery to the carcinoma cells and tissues

To summarize, nanotechnology gives more opportunities to use the drug with proven anticancer effects, but its application is significantly restricted by limited solubility, recrystalization upon dilution, and cosolvent-induced toxicity.

## Figures and Tables

**Table 1:** The effect of paclitaxel nanoformulation against different types of cancer.

Paclitaxel nanoformulation	Target tissue/type of cancer	Mode of delivery	Reference
Nanoformulated paclitaxel and AZD9291	Lung cancer	AZD9291-loaded disulfide cross-linking micelles (DCMs)	[94]
Low-dose nanoparticle albumin-bound paclitaxel (nab-PTX)	HER2-negative metastatic breast cancer	Albumin nanoparticles	[95, 96]
Nab-paclitaxel–gemcitabine combination	Pancreatic cancer	Albumin nanoparticles	[97]
Milk-derived exosomal formulation of PAC (ExoPAC)	Lung cancer	Exosomes	[98]
PTX-GemC12-LNC formulation	Brain tumour (glioblastoma)	Liquid nanoparticle	[99]
Paclitaxel-loaded PCL-TPGS nanoparticles: PTX-loaded poly(caprolactone)–alpha-tocopheryl polyethylene glycol 1000 succinate (PCL-TPGS) NPs	Breast cancer	PCL-TPGS copolymer	[100]
Paclitaxel-loaded poly(glycolide-*co*-*ε*-caprolactone)-*b*-D-*α*-tocopheryl polyethylene glycol 2000 succinate nanoparticles (PTX-loaded (PGA-*co*-PCL)-*b*-TPGS2k	Lung cancer	Amphiphilic copolymer (PGA-*co*-PCL)-*b*-TPGS2k	[101]
Paclitaxel and superparamagnetic iron oxide-loaded PEGylated poly(lactic-*co*-glycolic acid) (PLGA)-based nanoparticles (PTX/SPIO-NPs)	Glioblastoma	Superparamagnetic iron oxide-PEGylated poly(lactic-*co*-glycolic acid)-based nanoparticles	[102]
PTX micelles	Pulmonary carcinoma	Poly(ethylene glycol)-poly(3-caprolactone) copolymers (MPEG-PCL micelles)	[103]
Paclitaxel-loaded vitamin E-TPGS nanoparticles	Lung cancer Breast cancer Colorectal cancer Brain cancer Prostate cancer	TPGS nanoparticles	[75, 100, 104–107]
PTX nanomicelles using poly(styrene-*co*-maleic acid) (SMA); SMA-PTX micelles	Murine orthotopic colon cancer model	Poly(styrene-*co*-maleic acid)	[108]
Lumbrokinase/paclitaxel/poly(ethylene glycol)-*b*-(poly(ethylenediamine l-glutamate)-*g*-poly(*ε*-benzyoxycarbonyl-l-lysine)-*r*-poly(l-lysine)) LK/PTX/PEG-*b*-(PELG-*g*-(PZLL-*r*-PLL))	Bladder cancer	Poly(ethylene glycol)-*b*-(poly(ethylenediamine l-glutamate)-*g*-poly(*ε*-benzyoxycarbonyl-l-lysine)-*r*-poly(l-lysine))	[109]
Paclitaxel dimer-methoxypoly(ethylene glycol)*2K*-block-poly(D,L-lactide)*2K* (PTX dimer)/PEG-PDLLA formulations	Cervical cancer	Methoxypoly(ethylene glycol)*2K*-block-poly(D,L-lactide)*2K* (PEG-PDLLA) micelles	[110]
PTX-loaded redox-sensitive HSV nanoparticles	Lung cancer	Hyaluronic acid-disulfide-vitamin E succinate (HA-SS-VES, HSV) conjugate	[111]
Thermostable RNA-PTX nanoparticles (4WJ-X-24 PTX nanoparticles)	Breast cancer	RNA nanoparticles	[112]
